# The Dynamics of COVID-19 in Hiroshima Prefecture Compared to Japan and Its Association With Meteorological Factors: A Comparative Analysis

**DOI:** 10.7759/cureus.57708

**Published:** 2024-04-06

**Authors:** Md Razeen Ashraf Hussain, Syeda Sabrina Easmin Shaba, E. Bunthen, Kaniz Fateema Eity, Md Marufur Roshid, Md Abdul Kuddus

**Affiliations:** 1 Epidemiology and Research, Asian Institute of Disability and Development, Dhaka, BGD; 2 Epidemiology and Public Health, National Liver Foundation of Bangladesh, Dhaka, BGD; 3 Public Health and Health Policy, Graduate School of Biomedical and Health Sciences, Hiroshima University, Hiroshima, JPN; 4 Epidemiology and Public Health, National Payment Certification Agency, Ministry of Economic and Finance, Phnom Penh, KHM; 5 Oncology, Khwaja Yunus Ali Medical College and Hospital, Sirajgonj, BGD; 6 Psychosocial Rehabilitation, Graduate School of Biomedical and Health Sciences, Hiroshima University, Hiroshima, JPN; 7 Mathematics, University of Rajshahi, Rajshahi, BGD

**Keywords:** hiroshima, association, meteorological factors, dynamic, covid-19

## Abstract

Introduction

Despite the implementation of countermeasures and mass vaccination programs, the COVID-19 pandemic incidence was a vital public health concern. This study aimed to explore the dynamics of COVID-19 cases and assess the association of COVID-19 pandemic epidemiological data with meteorological factors in Hiroshima Prefecture compared to Japan.

Methods

We analyzed COVID-19 pandemic data in Japan's Hiroshima Prefecture from January 16, 2020, to May 9, 2023. Meteorological factors were examined at different time frames, and Spearman correlation coefficients were calculated for COVID-19 variables and variants based on GISAID whole genome analysis.

Results

Hiroshima Prefecture reported 816,788 COVID-19 cases and 1,371 fatalities, with a city-to-rural case ratio of 0.97:1. Infection rates were 17.42% for Japan and 15.83% for Hiroshima. Gender-wise, the ratio was 99:1, and the 30-39 age group in Hiroshima had the highest cases (15.5%). Among all meteorological factors, daily and 14-day average wind speed showed a weak correlation with incidence (-0.1954, P < 0.01; 0.3669 P < 0.01), fatalities (-0.1148, P < 0.01; -0.2232 P < 0.01), and incidence rate (-0.2042, P < 0.01; -0.3751, P < 0.01), respectively. Clade GRA was most frequent (39.7%), and among 61 variants, B.1.1.7, AY.29, and BA.1.1.2 were predominant. Precipitation was associated significantly with the Alpha variant (0.3373, P<0.01), while the Delta variant (0.2934, <0.05) weakly correlated with humidity.

Conclusion

COVID-19 pandemic trends in Hiroshima Prefecture paralleled Japan's, yet with lower incidence and fatalities compared to most prefectures. Significant associations were found between meteorological factors and COVID-19 metrics, including incidence, fatalities, incidence rate, and mutations in Hiroshima.

## Introduction

On 31st of December 2019, China reported 27 cases of an epidemic with unknown etiology in Wuhan City of Hubei Province [1]. On 11th February 2020, this epidemic was officially named Coronavirus disease- 2019 (COVID-19) and was acknowledged as an infectious disease caused by the SARS-CoV-2 virus, as it quickly spread within China and to a further 24 countries [2]. Till the 11th of March 2020, more than 118,000 cases in 114 countries and 4,291 people lost their lives, and the World Health Organization (WHO) declared COVID-19 a pandemic [3]. Researchers confirmed that the COVID-19-causing virus SARS-CoV-2 belongs to the Betacoronavirus genus and Coronaviridae family from where SARS-CoV and MERS-CoV belong [4,5]. It was also reported previously in research that SARS-CoV and MERS-CoV respond to a variation of meteorological factors [6,7]. Researchers also found that like other coronaviruses (CoVs), it causes cough, fever, respiratory disorder, and, in the worst-case scenario, kidney failure, pneumonia, and even death due to COVID-19 [8]. It had also been identified for a long time that respiratory diseases like pneumonia’s morbidity and mortality rate were highly correlated with changing weather variables [9]. 

Several studies found that meteorological factors such as humidity, temperature, and wind speed are classified as the top predictors of COVID-19 illness [10-12]. The association implied that meteorological factors played a role in the increase in COVID-19 incidence and fatalities, which needs to be investigated. However, the effect of meteorological factors on COVID-19 has not been established with conviction. In Japan, the first COVID-19 case was identified 16th of January 2020 [13]. Until May 2023, in these three years, more than 20 million cases were confirmed as COVID-19 positive in total all the prefectures of Japan. In Hiroshima Prefecture of Japan about 0.4 million [14]. There were plenty of countermeasures such as lockdowns and mass vaccination programs for five rounds at the residence [15]. Taking all the above into consideration, considering three and half years of epidemiological data and meteorological factors association would be insightful additional and valuable information. This study aimed to find the dynamics of COVID-19 cases in Hiroshima Prefecture compared with all other prefectures in Japan and the association of COVID-19 epidemiological data with meteorological factors.

## Materials and methods

Study settings and period

In this study, we determined the impact of weather factors on COVID-19 incidence, the incidence rate per 100,000, and fatalities with meteorological factors, specifically temperature (average, maximum, and minimum), humidity, wind speed, daylight, and precipitation. The investigation spanned approximately 3.5 epidemiological years, covering the period from 16 January 2020 to 09 May 2023, within the context of the COVID-19 pandemic in Hiroshima. This ecological study focused on Japan, situated within latitudes 26° N to 43° N and longitudes 127° E to 141° E in the Western Pacific Region, comprising a total of 47 prefectures. Hiroshima Prefecture is the second largest prefecture in the Chugoku region situated at 34°23′29″N 132°27′07″E with about 2.8 million population and 8480 square kilometers (km^2^) of area.

Data collection

In this comparative analysis, we collected day-by-day COVID-19 cases from open-source data provided by the Ministry of Health, Labor, and Welfare (MHLW) and the Hiroshima Prefecture's government office sites. We collected the daily cases report, case reports based on gender in Japan as well as Hiroshima and the case’s location in Hiroshima [14]. Meteorological data, encompassing maximum temperature (°C), average temperature (°C), minimum temperature (°C), relative humidity (%), wind speed (km/h), daylight hours, and precipitation, was obtained from the Japan Meteorological Agency website (https://www.jma.go.jp/jma/indexe.html). We collected daily average, 7-day average, and 14-day average meteorological data as COVID-19 was reported to incubate for 7-14 days [16]. COVID-19 whole genome analysis was conducted using reference genome sequences from the website of GISAID (https://www.gisaid.org/). We stored all the data in an Excel spreadsheet. Two independent co-authors (MRAH and SSES) performed the data collection separately, and any dispute was discussed. If no decision was obtained, a third co-author (MAK) was consulted to reach a consensus.

Mutational analysis and genome of COVID-19 variants

Whole-genome sequences of COVID-19 variants in Hiroshima were analyzed using Chromas 2.6.5 and sequence homology was determined through the nucleotide BLASTn program on the National Center for Biotechnology Information's (NCBI's) website. Multiple sequence alignment was conducted using the ClustalW Multiple Alignment algorithm in BioEdit 7.2.6 software (Informer Technologies, Los Angeles, USA), comparing selected genomes to the reference strain (NC_045512/Wuhan-Hu-1) and Japanese SARS-CoV-2 isolates. Clades were defined based on specific markers following the GISAID system.

Statistically analysis

The descriptive analysis was performed for the comparison of COVID-19 cases in Japan and Hiroshima. We approached the Spearman correlation coefficient between COVID-19 daily incidence, incidence rate, and fatalities with meteorological factors (Temperature, relative humidity, wind speed, daylight hours, and precipitation) [17]. The number of COVID-19 daily incidences was the independent variable whereas the daily average of meteorological factors was the dependent variable. Additionally, associations between host factors and mutation frequency, as well as weather parameters and mutational events of SARS-CoV-2, were determined using Spearman's rank correlation coefficient (r_s_). The following equation was employed to calculate the coefficient in this study:

r_s_=1-6{(∑d_i_^2^ )/(n(n^2^-1))}

Here, ‘n’ represents the number of observations, ‘di’ represents the difference between the ranks of observations, and represents the Spearman's correlation coefficient. Microsoft Excel for Microsoft 365 MSO (Microsoft Corporation, Redmond, USA) was used to store survey responses. Statistical analyses were conducted using JMP15.0.0 software (SAS Institute Inc., Cary, USA).

Ethical consideration

This study examined openly accessible data, and the datasets utilized underwent prior de-identification and complete anonymization. The analysis of publicly available data without any patient-identifying information did not necessitate ethical approval. The study was conducted in adherence to the principles outlined in the Declaration of Helsinki.

## Results

Distribution of COVID-19 cases and dataset

The dataset encompassed 33,738,398 cases and 74,085 fatalities reported from 47 Japanese prefectures till our study period. The average daily newly confirmed cases numbered 27,906.04 (range: 0-261,735), the mean fatalities were 61.28 (range: 0-491), and the average incidence rate per 100,000 across all prefectures was 21.99 (range: 0.00-206.9) in Japan. Specifically for the Hiroshima Prefecture, the dataset included 816,788 cases and 1,371 fatalities reported during the same study period. The mean daily newly confirmed cases for Hiroshima were 675.59 (range: 0-491), the mean fatalities were 1.13 (range: 0-17), and the average incidence rate per 100,000 across Hiroshima Prefecture was 24.13 (range: 0.00-313.4). (Table 1)

**Table 1:** Distribution of COVID-19 cases and dataset

	Total Cases (N)	Daily Cases (Mean)	Average Incidence Rate (Per 100000)	Total Fatalities (N)	Daily Fatalities (Mean)
Japan (All Prefectures)	33738398	27906.04	21.99	74085	61.28
Hiroshima	816788	675.59	24.13	1371	1.13

For meteorological factors, the mean of daily average temperature was 16.51 ℃ (range: -1.6-31.9℃), mean of daily maximum temperature was 21.15℃ (range: 2.1-38℃), mean of daily minimum temperature was 13℃ (range: -5-28℃), relative humidity was 67% (range: 33-95%), wind speed was 3.0 m/s (range: 1.2-8.8 m/s), daylight was 5.9 (range: 0-13.8) and precipitation was 4.9mm (range:0-205.5mm) in Hiroshima Prefecture.

Distribution of frequency of cases, age, and genders with COVID-19

**Figure 1:**
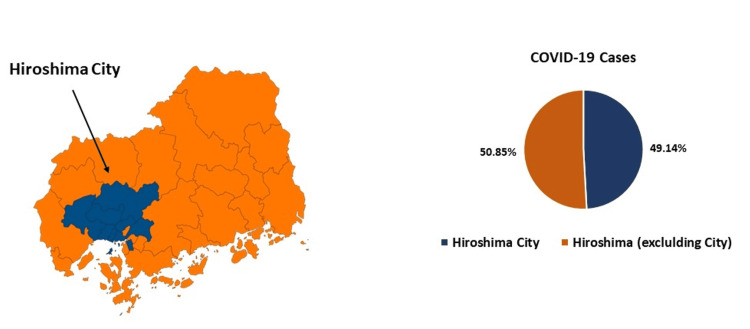
Population and COVID-19 case percentage in Hiroshima City and Hiroshima Prefecture (Excluding Hiroshima City) This figure shows the map of Hiroshima Prefecture and cases in Hiroshima City and Hiroshima Prefecture (excluding Hiroshima City). Orange color represents other than Hiroshima Prefecture excluding Hiroshima City and cases and blue color represents Hiroshima City and cases.

Details of COVID-19 cases in Hiroshima were published until October 6, 2022. The total cases up to that date were N=434,421, with 213,508 in Hiroshima City and 220,913 cases excluding Hiroshima City (Table 2). The case ratio between Hiroshima City and areas excluding it was 0.97:1 (Figure 1).

**Table 2:** Hiroshima Prefecture population, area, density and COVID-19 cases

Place	Population Density ( Per Square Kilometer)	Area (Square Kilometer)	Total Population (N)	COVID-19 Cases (N)
Hiroshima City	1324/km^2^	907 km^2^	1200754	213508
Hiroshima (Hiroshima City Excluded)	211.1/km^2^	7573 km^2^	1598948	220913
Hiroshima Prefecture	330.2/km^2^	8480 km^2^	2799702	434421

**Figure 2:**
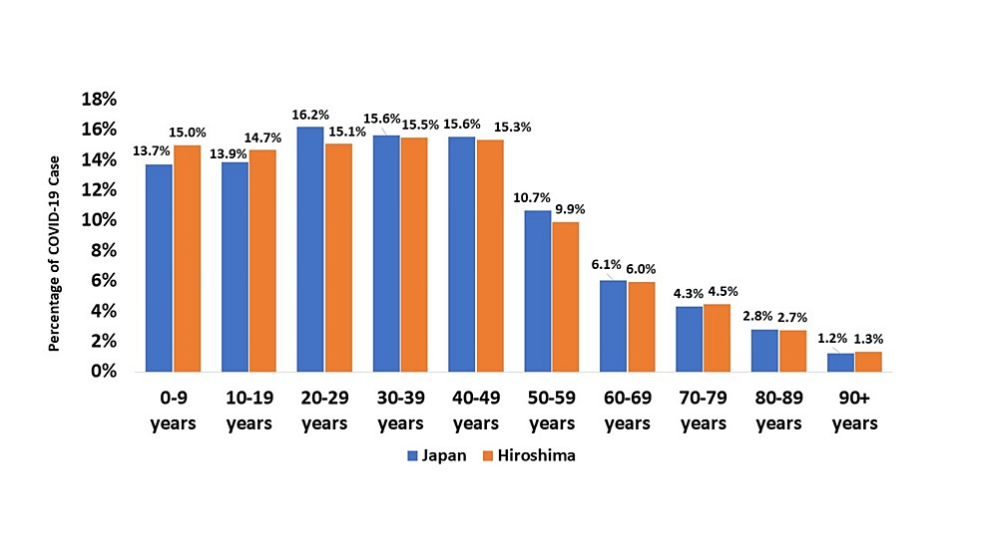
COVID-19 cases based on age distribution in Japan and Hiroshima This figure represents the age distribution of reported cases of COVID-19 in Japan and Hiroshima in percentage. In the figure, blue color represents cases of Japan and orange color represents the cases of Hiroshima Prefecture.

Approximately 17.42% (N=21460295) of the total Japanese population was infected with COVID-19, while 15.83% (N=434421) of the total population of Hiroshima Prefecture was affected. In terms of gender, the case ratio was 99:1 (Male: Female), and this pattern was consistent across all prefectures of Japan and Hiroshima. Regarding age distribution, the highest percentage of cases in Japan was in the 20-29 age group (16.2%, N=2948592), whereas in Hiroshima, the 30-39 age group had the highest number of cases (15.5%, N=64151). Interestingly, the <10 years age group had a higher percentage in Hiroshima (15%, N=62049) compared to all prefectures in Japan (13.7%, N= 2494076). Otherwise, the distribution was similar in Hiroshima and all the prefectures in Japan (Figure 2).

Correlation analysis between weather and the COVID-19 pandemic in Japan

**Figure 3:**
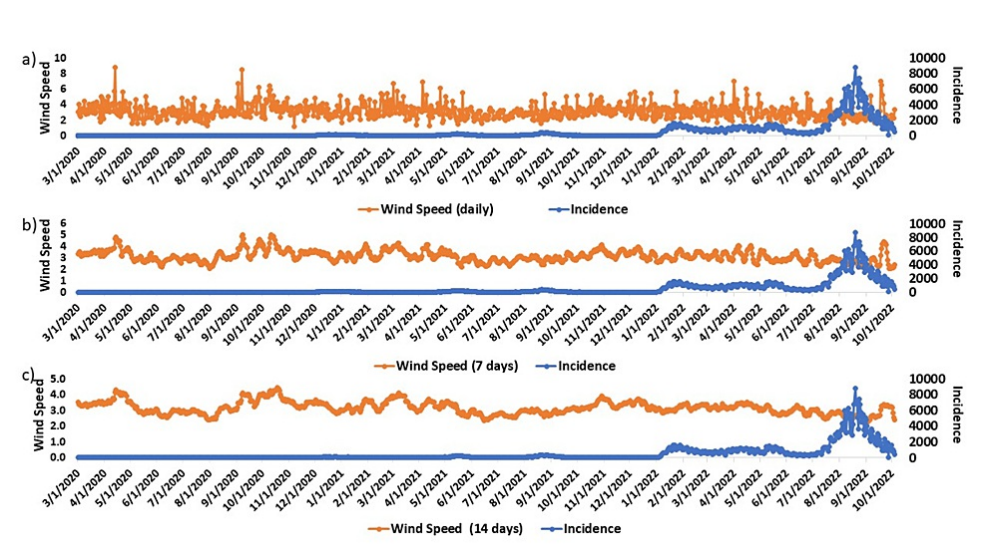
Association of wind speed - a)wind speed daily, b) wind speed 7 days, and c) wind speed 14 days - with COVID-19 incidence This figure demonstrates wind speed association with COVID-19 incidence. a) represents wind speed, b) represents 7 days of wind speed, and c) shows the association of 14 days of wind speed of association with incidence. Orange color represents wind speed and blue color represents incidence.

Spearman's correlation analysis was employed to assess the influence of weather factors on COVID-19 outcomes. Seven meteorological factors were examined across three timeframes: on the day of incidence, 7 days after, and 14 days after incidence in this study. The COVID-19 outcomes considered were incidences, fatalities, and the daily incidence rate per 100,000 population. The Spearman's rank correlation revealed correlations ranging from very weak to weak between each meteorological factor and the outcomes of COVID-19. Among meteorological factors, daily wind speed showed the highest correlation with incidence, fatalities, and incidence rate, with values of -0.1954 (P < 0.01), -0.1148 (P < 0.01), and -0.2042 (P < 0.01), respectively (Figure 3).

**Figure 4:**
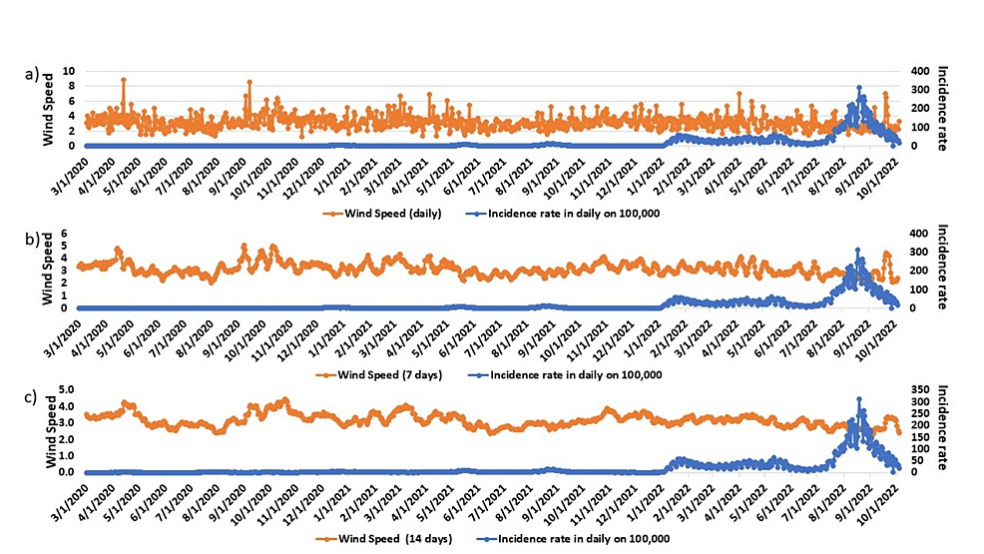
Association of wind speed - a) daily, b) 7 days, and c) 14 days - with COVID-19 incidence rate This figure demonstrates wind speed's association with the COVID-19 incidence rate. a) represents wind speed, b) represents 7 days wind speed, and c) shows the association of 14 days wind speed association with incidence rate. Orange color represents wind speed and blue color represents incidence.

The average wind speed for 7 days and 14 days demonstrated even higher correlations with incidence, fatalities, and incidence rate, yielding values of -0.3087 (P < 0.01), -0.1773 (P < 0.01), -0.3171 (P < 0.01), and -0.3669 (P < 0.01), -0.2232 (P < 0.01), -0.3751 (P < 0.01), respectively (Figure 4).

The daily average temperature was significantly correlated with incidence (0.1075, P < 0.01), fatalities (-0.0723, P < 0.05), and incidence rate (0.1068, P < 0.01). Additionally, 14-day average daylight hours were also significantly and negatively correlated with incidence (-0.1242, P < 0.01), fatalities (-0.117, P < 0.01), and incidence rate (0.129, P < 0.01). Other meteorological factors including relative humidity, daylight hours, and precipitation demonstrated very weak correlations with COVID-19 incidence, fatalities, and incidence rate (Table 3).

**Table 3:** COVID-19 incident, fatalities and incident rate Spearman association with meteorological factors p-value is considered significant and symbolized  ** as p-value<0.01, * as p-value <0.05

Period	Parameters	Spearman's coefficient for COVID-19 Incidence	Spearman's coefficient for COVID-19 Fatalities	Spearman's coefficient on incidence rate in daily on 100000
Daily Average	Temperature	0.1075**	-0.0723*	0.1068**
	Maximum Temperature	0.1062**	-0.0676*	0.105**
	Minimum Temperature	0.1071**	-0.0749*	0.1069**
	Humidity	0.0205	-0.0014	0.028
	Wind Speed	-0.1954**	-0.1148**	-0.2042**
	Day Light	-0.0312	-0.0323	-0.0375
	Precipitation	-0.0115	-0.0144	-0.0049
7 Days Average	Temperature	0.0971**	-0.0846**	0.0957**
	Maximum Temperature	0.0978**	-0.0813*	0.0961**
	Minimum Temperature	0.0979**	-0.0855**	0.0964**
	Humidity	0.0213	-0.0401	0.0275
	Wind Speed	-0.3087**	-0.1773**	-0.3171**
	Day Light	-0.0947**	-0.0553	-0.1024**
	Precipitation	-0.0384	-0.048	-0.032
14 Days Average	Temperature	0.0934**	-0.0886**	0.0917**
	Maximum Temperature	0.0928**	-0.0863**	0.0911**
	Minimum Temperature	0.0884**	-0.0912**	0.0866**
	Humidity	0.0246	-0.0298	0.0289
	Wind Speed	-0.3669**	-0.2232**	-0.3751**
	Day Light	-0.1242**	-0.117**	-0.129**
	Precipitation	-0.0278	-0.0751*	-0.0239

SARS-CoV-2 clade and variant distributions during the COVID-19 pandemic

**Figure 5:**
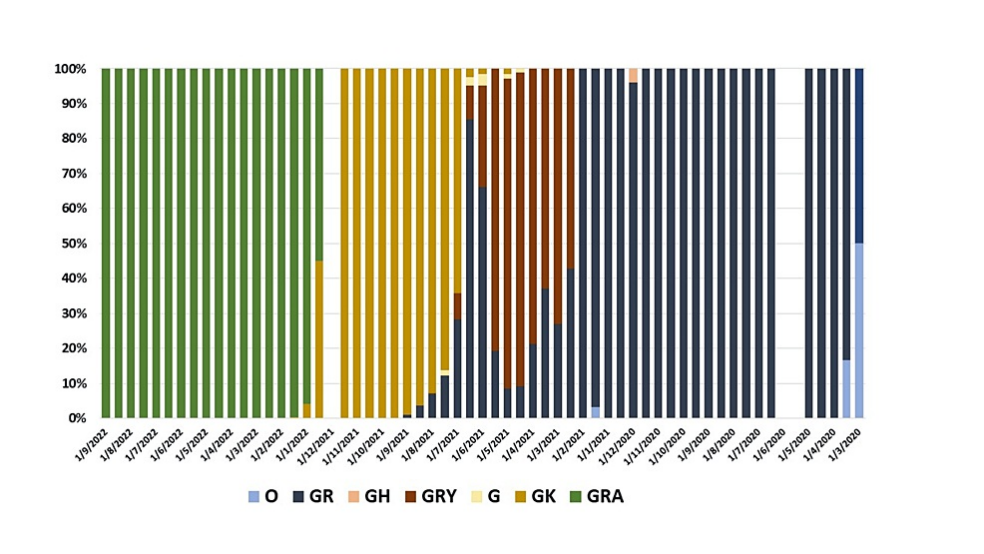
Distribution of clade from the submitted sample of COVID-19 in Hiroshima This figure represents the distribution of clade in percentage (%) from analyzed COVID-19 cases in Hiroshima from March 2020 to September 2022. Blue color represents the O clade, grey color represents the GR clade, orange color represents the GH clade, brown color represents the GRY clade, yellow color represents the G clade, gold color represents the GK clade and green color represents the GRA clade.

Approximately 5,520 full genomes of COVID-19 from Hiroshima Prefecture were analyzed until October 2022. The temporal distribution of clades and variants was determined, with clade GRA being the most frequent (39.7%, N=2194), followed by GK (22.3%, N=1233), GRY (18.7%, N=1031), G (0.3%, N=15), GH (0.1%, N=6), and O (0.1%, N=3) (Figure 5).

The GR clade was also detected from March 2020 to February 2021, followed by GRY from March 2021 to June 2021. The highest frequencies of GK and GRA were observed from July 2021 to November 2021 and December 2021 to September 2022. A total of 61 variants of lineages were identified, with B.1.1.7 (22.9%, N=1264), AY.29 (20.6%, N=1137), and BA.1.1.2 (20.1%, N=1110) being predominant among all lineages.

Correlation analysis between mutation frequency and weather parameters

Utilizing genetic codes and mutations, we categorized variants into Alpha, Delta, Omicron, and others following CDC guidelines [18]. Considering detection and sample collection onset, we examined the association between variants and meteorological factors. Precipitation showed a significant association with the Alpha variant (0.3373, P<0.01). In contrast, the Delta variant exhibited a weak association with humidity (0.2934, <0.05), and other variants showed a weak association with wind speed (0.3445, P<0.01). No significant association was observed with the remaining variants (Table 3).

**Table 4:** COVID-19 variant association Spearman association with meteorological factors in Hiroshima p-value is considered significant and symbolized  ** as p-value<0.01, * as p-value <0.05

Parameters	Alpha	Delta	Omicron	Others
Average Temperature	0.1374	0.1486	-0.0438	-0.2374
Maximum Temperature	0.1346	0.1546	-0.051	-0.2347
Minimum Temperature	0.1315	0.1484	-0.0541	-0.2254
Humidity	0.1	0.2934*	-0.1135	-0.2031
Wind Speed	-0.1309	-0.1615	-0.2294	0.3445**
Day Light	-0.0478	-0.2294	0.006	0.0849
Precipitation	0.3373**	0.0684	-0.1465	-0.0808

## Discussion

Numerous factors including host health conditions, direct man-to-man transmission, gatherings of people, comorbidity, coinfection, age, gender, the exportation of new variants, and meteorological parameters regulated the transmission rate and outcome of the pandemic. Our study revealed the association of meteorological parameters with 3.5 years of COVID-19 epidemiological data in local settings in Hiroshima, Japan. Our study also highlighted that meteorological factors are significantly associated with COVID-19 incidence, fatalities rate, and incidence rate.

Our study found that based on gender female cases were slightly higher than male. Another similar study also found similar results [19]. The case ratio between the city and other area cases was similar to our findings. However, other studies found a higher ratio in city areas than in rural areas [20,21]. The probable reason might be the long study period, mass vaccination, and unparalleled awareness program of those studies. Regarding the age distribution, the highest percentage of cases in Japan was in the 20-29 years age group whereas in Hiroshima, the 30-39 years age group had the highest number of cases. However, most of the studies found the 20-29 years age group has the highest number of cases [22,23]. In our findings, the <10 years age group had a higher percentage of cases in Hiroshima compared to all prefectures in Japan. The probable reason could be the proportion of vaccination and precautions [24].

In our study, we found correlations that range from very weak to weak between each of the meteorological factors and the outcomes of COVID-19. Several studies found similar associations between meteorological factors with COVID-19 incidence, fatalities, and incidence rate [25]. Our study found weak associations between wind speed, temperature, relative humidity, precipitation, and daylight hours with COVID-19 incidence, fatalities, and incidence rate which is similar to other studies [26-28]. We analyzed full genomes of COVID-19 from Hiroshima Prefecture over approximately three years to examine different variants and lineages. In our findings, clade GRA was mostly associated with the Alpha variant, clade GRY was predominantly found in the Delta variant, and GRA represented the Omicron variant. Other lineages of variants had much lower percentages; hence, they were not mentioned.

Considering detection and sample collection onset, we examined the association between variants and meteorological factors. Precipitation showed a significant association with the Alpha variant, the Delta variant exhibited an association with humidity and other variants showed an association with wind speed. Similar findings were also observed in other studies [29,30]. These findings highlight the importance of considering environmental factors alongside genomic surveillance to better understand and mitigate the spread of SARS-CoV-2 variants.

Our study observed approximately 3.5 years of epidemiological data. To the best of our knowledge, this is the first study that included such a long period of COVID-19 data for Hiroshima, Japan. Our study found similarities in cases based on age and gender compared to all other prefectures in Japan. The findings revealed a weak association of COVID-19 incidence, fatalities, and incidence rate with the meteorological factor of wind speed. We also found significant associations with the Alpha, Delta, and other variants with meteorological factors.

There were several limitations in our study. COVID-19 transmission can be influenced by various factors such as crowd density, mobility, vaccination rates, and others, which we did not consider in our study. Secondly, we obtained the data from the Ministry of Health, Labour and Welfare of Japan. There might be some missing data for the entire country regarding incidence, fatalities, and incidence rate. Thirdly, meteorological data might vary across different locations within Hiroshima Prefecture. We calculated the mean of meteorological factors for the entire prefecture of Hiroshima.

## Conclusions

A notable correlation was observed between meteorological factors and COVID-19 incidence, fatalities, incidence rate, and mutations. Wind speed demonstrated the strongest association among all meteorological factors with COVID-19 data. The COVID-19 dynamics in Hiroshima Prefecture mirrored those of Japan, although the incidence and fatalities in Hiroshima were considerably lower than in most other prefectures of Japan. The major variants in Hiroshima included Alpha, Delta, and Omicron. Additionally, an association between variants and meteorological factors was identified in Hiroshima prefecture. This study suggests that meteorological factors, along with preventive measures and interventions, play significant roles in shaping the outcomes and severity of the COVID-19 pandemic. Further in-depth studies are warranted. The findings from our study can contribute to international health policymakers' understanding of the COVID-19 pandemic, aiding in the formulation of appropriate measures to minimize its impact in the future.

## References

[1] (2024). Chinese officials probe unidentified pneumonia outbreak in Wuhan. https://www.cidrap.umn.edu/news-perspective/2019/12/news-scan-dec-31-2019..

[2] Cucinotta D, Vanelli M (2020). WHO declares COVID-19 a pandemic. Acta Biomed.

[3] (2024). WHO Director-General's opening remarks at the media briefing on COVID-19 - 11 March. https://www.who.int/director-general/speeches/detail/who-director-general-s-opening-remarks-at-the-media-briefing-on-covid-19---11-march-2020..

[4] Pal M, Berhanu G, Desalegn C, Kandi V (2020). Severe acute respiratory syndrome coronavirus-2 (SARS-CoV-2): an update. Cureus.

[5] Petrosillo N, Viceconte G, Ergonul O, Ippolito G, Petersen E (2020). COVID-19, SARS and MERS: are they closely related?. Clin Microbiol Infect.

[6] Hu B, Guo H, Zhou P, Shi ZL (2021). Characteristics of SARS-CoV-2 and COVID-19. Nat Rev Microbiol.

[7] Dhama K, Khan S, Tiwari R (2020). Coronavirus disease 2019-COVID-19. Clin Microbiol Rev.

[8] Ali I, Alharbi OM (2020). COVID-19: Disease, management, treatment, and social impact. Sci Total Environ.

[9] Davis RE, Rossier CE, Enfield KB (2012). The impact of weather on influenza and pneumonia mortality in New York City, 1975-2002: a retrospective study. PLoS One.

[10] Topaloglu MS, Sogut O, Az A, Ergenc H, Akdemir T, Dogan Y (2023). The impact of meteorological factors on the spread of COVID-19. Niger J Clin Pract.

[11] Chen S, Huang L, Cai D, Li B, Yang J (2023). Association between meteorological factors and COVID-19: a systematic review. Int J Environ Health Res.

[12] Hridoy AE, Mohiman MA, Tusher SM, Nowraj SZ, Rahman MA (2021). Impact of meteorological parameters on COVID-19 transmission in Bangladesh: a spatiotemporal approach. Theor Appl Climatol.

[13] (2024). Current status of new coronavirus infection and response by the Ministry of Health, Labor and Welfare. https://www.mhlw.go.jp/stf/covid-19/kenkou-iryousoudan_00006.html.

[14] (2024). Hiroshima Prefectural Center for Disease Control and Prevention. https://www.pref.hiroshima.lg.jp/site/hcdc/covid19-kanjya.html..

[15] Ma M, Shi L, Liu M, Yang J, Xie W, Sun G (2023). Comparison of COVID-19 vaccine policies and their effectiveness in Korea, Japan, and Singapore. Int J Equity Health.

[16] Bikbov B, Bikbov A (2021). Maximum incubation period for COVID-19 infection: do we need to rethink the 14-day quarantine policy?. Travel Med Infect Dis.

[17] Mukaka MM (2012). Statistics corner: A guide to appropriate use of correlation coefficient in medical research. Malawi Med J.

[18] (2024). COVID-19, Variants of the Virus. https://www.cdc.gov/coronavirus/2019-ncov/variants/index.html..

[19] Doerre A, Doblhammer G (2022). The influence of gender on COVID-19 infections and mortality in Germany: insights from age- and gender-specific modeling of contact rates, infections, and deaths in the early phase of the pandemic. PLoS One.

[20] Tazerji SS, Shahabinejad F, Tokasi M (2022). Global data analysis and risk factors associated with morbidity and mortality of COVID-19. Gene Rep.

[21] Iyanda AE, Boakye KA, Lu Y, Oppong JR (2022). Racial/Ethnic heterogeneity and rural-urban disparity of COVID-19 case fatality ratio in the USA: a negative binomial and GIS-based analysis. J Racial Ethn Health Disparities.

[22] Monod M, Blenkinsop A, Xi X (2021). Age groups that sustain resurging COVID-19 epidemics in the United States. Science.

[23] Salman HM, Syed J, Riaz A, Sarfraz Z, Sarfraz A, Bokhari SH, Ojeda IC (2022). An epidemiological, strategic and response analysis of the COVID-19 pandemic in South Asia: a population-based observational study. BMC Public Health.

[24] (2024). Regarding the domestic outbreak of the new coronavirus infection, etc. https://www.mhlw.go.jp/stf/covid-19/kokunainohasseijoukyou.html..

[25] Islam MM, Noor FM (2022). Correlation between COVID-19 and weather variables: a meta-analysis. Heliyon.

[26] Guo C, Bo Y, Lin C (2021). Meteorological factors and COVID-19 incidence in 190 countries: an observational study. Sci Total Environ.

[27] Clouston SA, Morozova O, Meliker JR (2021). A wind speed threshold for increased outdoor transmission of coronavirus: an ecological study. BMC Infect Dis.

[28] Ogaugwu C, Mmaduakor C, Adewale O (2023). Association of meteorological factors with COVID-19 during Harmattan in Nigeria. Environ Health Insights.

[29] Tam NT, Anh NT, Tung TS (2023). Spatiotemporal evolution of SARS-CoV-2 Alpha and Delta variants during large nationwide outbreak of COVID-19, Vietnam, 2021. Emerg Infect Dis.

[30] Sobral MF, Duarte GB, da Penha Sobral AI, Marinho ML, de Souza Melo A (2020). Association between climate variables and global transmission oF SARS-CoV-2. Sci Total Environ.

